# Correction: Bauer et al. Challenges for Optimization of Reverse Shoulder Arthroplasty Part II: Subacromial Space, Scapular Posture, Moment Arms and Muscle Tensioning. *J. Clin. Med.* 2023, *12*, 1616

**DOI:** 10.3390/jcm12227004

**Published:** 2023-11-09

**Authors:** Stefan Bauer, William G. Blakeney, Allan W. Wang, Lukas Ernstbrunner, Jocelyn Corbaz, Jean-David Werthel

**Affiliations:** 1 Service d’Orthopédie et de Traumatologie, Chirurgie de l’Épaule, Ensemble Hospitalier de la Côte, 1110 Morges, Switzerland; 2 Medical School, University of Western Australia, 35 Sterling Highway, Perth, WA 6009, Australia; 3 Department of Orthopaedic Surgery, Royal Perth Hospital, Perth, WA 6000, Australia; 4 Department of Orthopaedic Surgery, Royal Melbourne Hospital, Parkville, VIC 3050, Australia; 5 Department of Biomedical Engineering, University of Melbourne, Parkville, Melbourne, VIC 3010, Australia; 6 Service d’Orthopédie et de Traumatologie, Centre Hospitalier Universitaire Vaudois, 1011 Lausanne, Switzerland; 7 Service d’Orthopédie et de Traumatologie, Hôpital Ambroise Paré, 9 Avenue Charles de Gaulle, 92100 Boulogne-Billancourt, France

In the original publication [1], three cited references in the main text were not listed in the last reference list, and two references were cited incorrectly and needed to be replaced. The missed references were inserted as references [12], [44] and [48]. The miscited references were revised as references [40] and [45].

With this correction, the order of some references was adjusted accordingly. Reference [37] in the main text was revised to [42], reference [42] was revised to [43], reference [43] was revised to [44], reference [44] in Section 4.1 was revised to [45], reference [44] in Section 4.2 was revised to [47], reference [45] in the first paragraph of Section 4.2 was revised to [46] and reference [45] in the last paragraph of Section 4.2 was revised to [48].

The authors state that the scientific conclusions are unaffected. This correction was approved by the Academic Editor. The original publication was also updated.

**References List**

12. Neeley, R.; Simon, P.; Christmas, K.N.; Gorman, R.A., II; Amador, I.E.; Frankle, M.A.; Mighell, M.A. Radiographic outcomes of patients undergoing reverse shoulder arthroplasty using inlay versus onlay components: Is there really a difference? *Semin. Arthroplast. JSES* **2021**, *31*, 620–628.
40. Kadum, B.; Wahlström, P.; Khoschnau, S.; Sjödén, G.; Sayed-Noor, A. Association of lateral humeral offset with functional outcome and geometric restoration in stemless total shoulder arthroplasty. *J. Shoulder Elb. Surg.* **2016**, *25*, e285–e294.
44. Mahendraraj, K.A.; Colliton, E.; Muniz, A.; Menendez, M.E.; Jawa, A. Assessing the validity of the distalization and lateralization shoulder angles following reverse total shoulder arthroplasty. *Semin. Arthroplast. JSES* **2020**, *30*, 291–296.
45. Di Giacomo, G. The Effect of Reverse Shoulder Arthroplasty Design Parameters on Rotator Cuff and, Deltoid Muscle Torques: A Computational Biomechanical Analysis. In Proceedings of the Advanced Shoulder Arthroplasty Meeting, Snowbird, UT, USA, 21 January 2022.
48. Schmalzl, J.; Fenwick, A.; Reichel, T.; Schmitz, B.; Jordan, M.; Meffert, R.; Plumhoff, P.; Boehm, D.; Gilbert, F. Anterior deltoid muscle tension quantified with shear wave ultrasound elastography correlates with pain level after reverse shoulder arthroplasty. *Eur. J. Orthop. Surg*. *Traumatol.* **2022**, *32*, 333–339.
